# AEG-1 Contributes to Metastasis in Hypoxia-Related Ovarian Cancer by Modulating the HIF-1alpha/NF-kappaB/VEGF Pathway

**DOI:** 10.1155/2018/3145689

**Published:** 2018-03-25

**Authors:** Xiaoyu Yu, Yan Wang, Huilei Qiu, Hongtao Song, Di Feng, Yang Jiang, Shuzhe Deng, Hongxue Meng, Jingshu Geng

**Affiliations:** Department of Pathology, Harbin Medical University Cancer Hospital, Harbin, China

## Abstract

**Objective:**

Ovarian carcinoma represents one of the deadliest malignancies among female cancer patients. Astrocyte-elevated gene-1 (AEG-1) participates in the ontogenesis of multiple human malignant diseases. Here we evaluated AEG-1, hypoxia-inducible factor- (HIF-) 1*α*, nuclear factor kappa-light-chain-enhancer of activated B cells (NF-*κ*B), and vascular endothelial growth factor (VEGF) amounts in hypoxia induced ovarian carcinoma cells. This study aimed to explore the mechanism by which AEG-1 regulates metastasis in hypoxia induced ovarian carcinoma.

**Patients and Methods:**

AEG-1, HIF-1*α*, and VEGF protein amounts were evaluated by immunohistochemistry in 40 and 170 normal ovary and ovarian cancer tissue specimens, respectively. In addition, AEG-1, HIF-1*α*, NF-*κ*B, and VEGF mRNA and protein levels were determined by reverse quantified RT-PCR and WB, respectively, at different time periods (0–24 h) in epithelial ovarian cancer (EOC) SKOV3 cells treated in a hypoxia incubator. Furthermore, NF-*κ*B and VEGF gene and protein expression levels in AEG-1 knockdown EOC cells were quantitated by RT-PCR and WB, respectively.

**Results:**

AEG-1, HIF-1*α*, and VEGF amounts were significantly elevated in EOC tissue samples compared with normal ovary specimens (*p* < 0.001). Positive expression of HIF-1*α* and AEG-1 was associated with higher metastatic rate (*p* < 0.01), lower FIGO stage (*p* < 0.001), and degree of differentiation (*p* < 0.001). Meanwhile, EOC SKOV3 cells grew upon exposure to hypoxia for 8 h (*p* < 0.001); at this time point, AEG-1, HIF-1*α*, NF-*κ*B, and VEGF amounts peaked (*p* < 0.001), at both the gene and the protein levels. After AEG-1 knockdown, HIF-1*α*, NF-*κ*B, and VEGF amounts were significantly decreased in EOC SKOV3 cells, also under hypoxic conditions (*p* < 0.01).

**Conclusions:**

As an independent prognostic factor, AEG-1 was found to be significantly associated with hypoxia in ovarian cancer by regulating the HIF-1alpha/NF-kappaB/VEGF pathway. Therefore, AEG-1 may be useful in determining disease stage and prognosis in ovarian cancer.

## 1. Introduction

Ovarian carcinoma, a commonly encountered primary malignancy, represents one of the deadliest malignant tumors in female patients around the world. Astrocyte-elevated gene-1 (AEG-1) plays multiple roles and acts as an important molecule regulating a variety of events in carcinogenesis. Mounting evidence indicates that AEG-1 expression is elevated in a wide range of malignancies [1, 2], such as hepatocellular, gallbladder, renal cell, breast, lung, and ovarian carcinomas [3]. The above works showed that AEG-1 constitutes a critical transcription factor in cancer metastasis and invasion. However, the mechanism by which AEG-1 regulates metastasis in ovarian carcinoma remains largely unknown.

HIF-1*α*, firstly identified as a transcription factor, is activated by hypoxic stress. In addition, it acts as a hypoxia-inducible nuclear factor in VSMCs and has significant functions in hypoxic responses in human cells, modulating the transcription of hypoxia-inducible genes [4].

This work evaluated AEG-1, HIF-1*α*, NF-*κ*B, and VEGF protein and mRNA amounts in epithelial ovarian cancer (EOC) cells cultured under hypoxic conditions and found potential associations of AEG-1, NF-*κ*B, and VEGF expression levels with hypoxia induced ovarian cancer growth.

## 2. Materials and Methods

### 2.1. Patient Population

The current study had approval from the Cancer Hospital of Harbin Medical University, Harbin, China. Written informed consent was obtained from each patient. Samples were collected from 170 and 40 patients with EOC and normal ovary suffering from other diseases, respectively, who underwent surgery between February 2007 and February 2009 in the Cancer Hospital of Harbin Medical University. Tumor stage in each patient was evaluated according to the International Federation of Gynecology and Obstetrics (FIGO) classification. Histopathological grades were determined according to the World Health Organization criteria.

### 2.2. Immunohistochemical Staining

AEG-1, HIF-1*α*, and VEGF protein levels were assessed immunohistochemically in biopsy samples after paraffin-embedding, by the avidin-biotin immunoperoxidase method, as instructed by the manufacturer. After dewaxing and rehydration by standard methods, the sections were incubated with primary antibodies targeting human AEG-1 (ab45338, Abcam), HIF-1*α* (ab16066, Abcam), and VEGF (ab155944, Abcam) overnight at 4°C, respectively, followed by incubation with biotin-conjugated secondary antibodies (Santa Cruz, USA). Negative control slides were incubated with rabbit serum in lieu of primary antibodies.

#### 2.2.1. Cell Culture

Human EOC SKOV3 cells were purchased from the Cell Institute of Chinese Academy of Sciences, Shanghai, and grown in McCoy's 5A medium containing 10% fetal bovine serum at 37°C in a normal 5% CO2 cell culture incubator. To induce hypoxia, cell culture was performed in an anaerobic chamber containing 1% O2, 5% CO2, and 94% N2, at 37°C for 0, 2, 4, 8, 10, 12, and 24 h, respectively.

#### 2.2.2. Lentiviral Infection

AEG-1 knockdown lentivirus was manufactured by Shanghai GenePharma Co.,

Ltd. SKOV3 cells were plated into 3.5 cm dishes (1 × 10^6^ cells/dish) for 24 h prior to lentiviral infection at a multiplicity of infection (MOI) of 4. Infection efficiency, assessed by fluorescence microscopy detecting GFP at 48 h after infection, was >90%.

#### 2.2.3. Quantified Real-Time PCR Assay

Cellular RNA extraction was carried out with TRIzol reagent (Takara, Otsu, Shi, Japan). Then, real-time PCR was performed on a DNA Engine Opticon™ sequence detector. Primers for AEG-1, HIF-1*α*, and VEGF were designed with Primer Bank. Mean Ct (triplicate experiments) was employed for data analysis, with the endogenous control U6 snRNA used for normalization.

#### 2.2.4. Immunoblot

Total protein was obtained from SKOV3 cells using RIPA buffer supplemented with proteinase/phosphatase inhibitors (Thermo, Cambridge, MA). Equal amounts of total protein were resolved by 10 or 12% SDS-PAGE, followed by transfer onto nitrocellulose membranes (Millipore, Bedford, MA). Anti-AEG-1 (1 : 500), NF-*κ*B (1 : 1000), HIF-1*α* (1 : 500), and VEGF (1 : 1000) (Abcam) primary antibodies were used for detection.

#### 2.2.5. Transwell Invasion Assay

Cells cultured in media with or without 100 nM rapamycin were harvested after 8 h of exposure to hypoxia. Those cultured under normoxic conditions in parallel were used as the control group.

### 2.3. Statistics

Data are mean ± SEM. Groups were compared by one-way analysis of variance (ANOVA) with SPSS 13.0 (SPSS, USA), followed by Bonferroni post hoc tests. Unpaired Student's* t*-test was used to assess group pairs. A statistical significance level of 0.05 was used.

## 3. Results

### 3.1. AEG-1, HIF-1*α*, and VEGF Levels in Ovarian Carcinoma and Noncancerous Specimens

**Figure 1:**
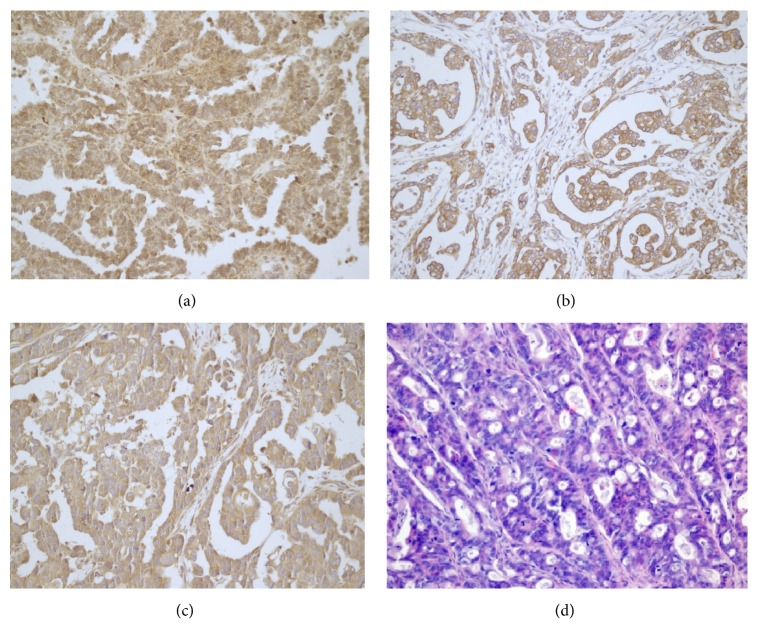
AEG-1, VEGF, and HIF-1*α* expression levels in ovarian carcinoma. (a) High AEG-1 expression; (b) high VEGF expression; (c) high HIF-1*α* expression; (d) H&E staining of (a)–(d) (×100).

By immunohistochemical staining, we first observed that AEG-1 was primarily localized in the cytosol of ovarian cancer cells. Meanwhile, HIF-1*α* was expressed in both cytoplasmic and nuclear compartments. VEGF was mainly expressed in the cytoplasmic compartment or cell membrane (Figure 1).

Assessing the expression levels of these three proteins in tumor samples and normal specimens, we found that 62.9%, 60%, and 54.7% ovarian cancer tissue specimens were positive for AEG-1, HIF-1*α*, and VEGF, respectively, for only 5%, 5%, and 10% obtained in normal ovarian tissue samples, respectively (all *p* < 0.001) (Table 1).

### 3.2. Associations of HIF-1*α* and AEG-1 Levels with Clinicopathologic Features of EOC Patients

AEG-1 expression in ovarian cancer specimens was significantly associated with histological type, metastasis, FIGO stage, and residual tumor but not correlated with age (Table 2). Similarly, HIF-1*α* levels were associated with histological type, metastasis, clinical stage, FIGO stage, and residual tumor (Table 2). Specifically, the expression levels of AEG-1 and HIF-1*α* were higher in stages III/IV than in stages I/II (Table 2), and the differences reached statistical significance (Table 2).

### 3.3. HIF-1*α*, VEGF, and AEG-1 Expression Levels Are Positively Correlated in Ovarian Carcinoma

Poor prognosis in ovarian cancer results from concerted effects of multiple genes. In this study, AEG-1 levels were obviously associated with HIF-1*α* and VEGF amounts (Table 3, all *p* < 0.001). Meanwhile, HIF-1*α* amounts were positively associated with VEGF and AEG-1 levels (Table 3, all *p* < 0.001).

### 3.4. Hypoxia Increases Invasion in Ovarian Carcinoma Cells

**Figure 2:**
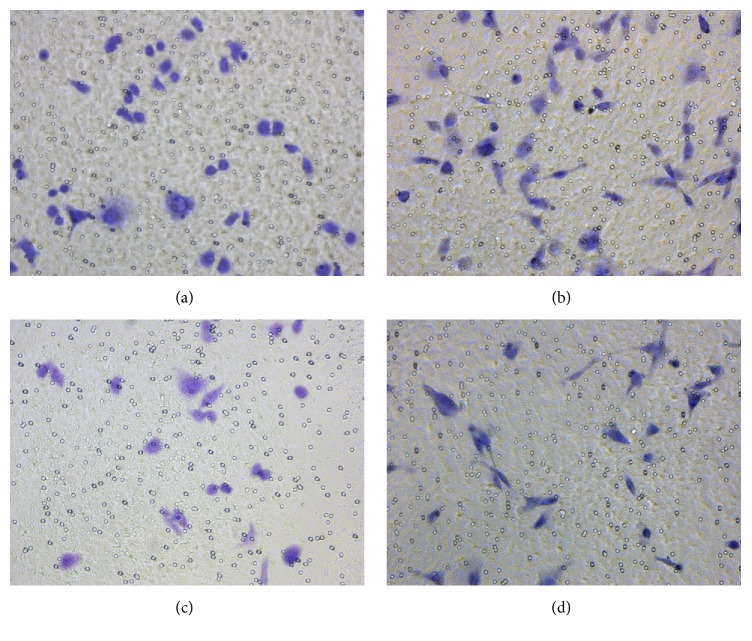
*Wild type and AEG-1 knockdown SKOV3 cells grown under hypoxic and normoxic conditions.* (a) SKOV3 cells in normoxia; (b) SKOV3 cells in hypoxia; (c) len-AEG-1 SKOV3 cells in normoxia; (d) len-AEG-1 SKOV3 cells in hypoxia. Data are mean ± SD from three independent experiments.

**Figure 3:**
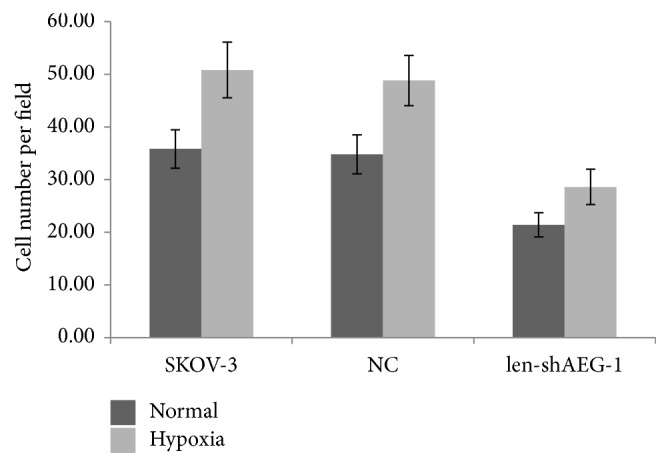
Hypoxia increases the invasive ability of the EOC cell line, which is reduced by AEG-1 silencing.

Transwell assays were employed to estimate the invasive ability of the EOC SKOV3 cell line. The results showed that the invasive ability of SKOV3 cells was significantly enhanced in hypoxia compared with normoxia (Figures 2 and 3). These findings suggested that hypoxia increased the invasive ability of EOC cells.

Interestingly, the invasive ability of AEG-1 knockdown SKOV3 cells was overtly reduced compared with that of wildtype SKOV3 cells, in both normoxic and hypoxic culture conditions (Figures 2 and 3). This indicated that AEG-1 might be a key effector of hypoxia induced ovarian cancer growth.

### 3.5. Hypoxia Upregulates AEG-1, HIF-1*α*, NF-*κ*B, and VEGF

As shown above, high AEG-1 expression was associated with metastasis in ovarian cancer (Table 2), and hypoxia could promote the invasive ability of cultured ovarian carcinoma cells (Figure 3). Furthermore, AEG-1 was involved in hypoxia induced ovarian cancer cell growth (Figure 3). Therefore, we hypothesized that hypoxia might affect AEG-1 expression.

**Figure 4:**
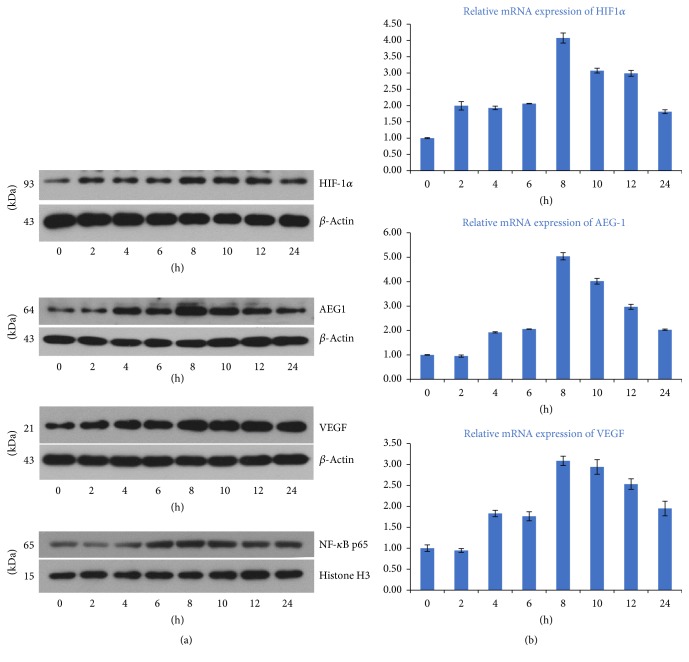
*AEG-1, VEGF, NF-κB, and HIF-1α expression levels in ovarian carcinoma SKOV3 cells cultured under hypoxic conditions at different time points (0, 2, 4, 8, 10, 12, and 24 h)*. (a) After culture under hypoxic conditions for 8 h, AEG-1, HIF-1*α*, NF-*κ*B, and VEGF protein amounts peaked (*p* < 0.001) as determined by Western blotting. (b) After culture under hypoxic conditions for 8 h, AEG-1, HIF-1*α*, and VEGF mRNA amounts peaked (*p* < 0.001) as determined by real-time PCR. Data are mean ± SD from three independent experiments.

SKOV3 cells were cultured under hypoxic conditions for different times (0, 2, 4, 6, 8, 10, 12, and 24 h), for total protein and RNA extraction. As expected, hypoxia treated SKOV3 cells showed markedly increased AEG-1 protein and mRNA levels, which peaked at 8 h of culture under hypoxic conditions (Figure 4, *p* < 0.01). These findings suggested that hypoxia upregulated AEG-1.

HIF-1*α* and VEGF levels were positively correlated with ovarian cancer metastasis, as shown above (Tables 1 and 2). Therefore, we detected level changes of HIF-1*α*, NF-*κ*B, and VEGF in SKOV3 cells cultured under hypoxic conditions, and similar results were found. Indeed, HIF-1*α*, NF-*κ*B, and VEGF amounts changed gradually with prolonged hypoxia (Figure 4, *p* < 0.01). Overall, our results showed that AEG-1, HIF-1*α*, NF-*κ*B, and VEGF transcription levels were altered by hypoxia.

### 3.6. Hypoxia Associated Upregulation of HIF-1*α*, VEGF, and NF-*κ*B Is Dependent on AEG-1

**Figure 5:**
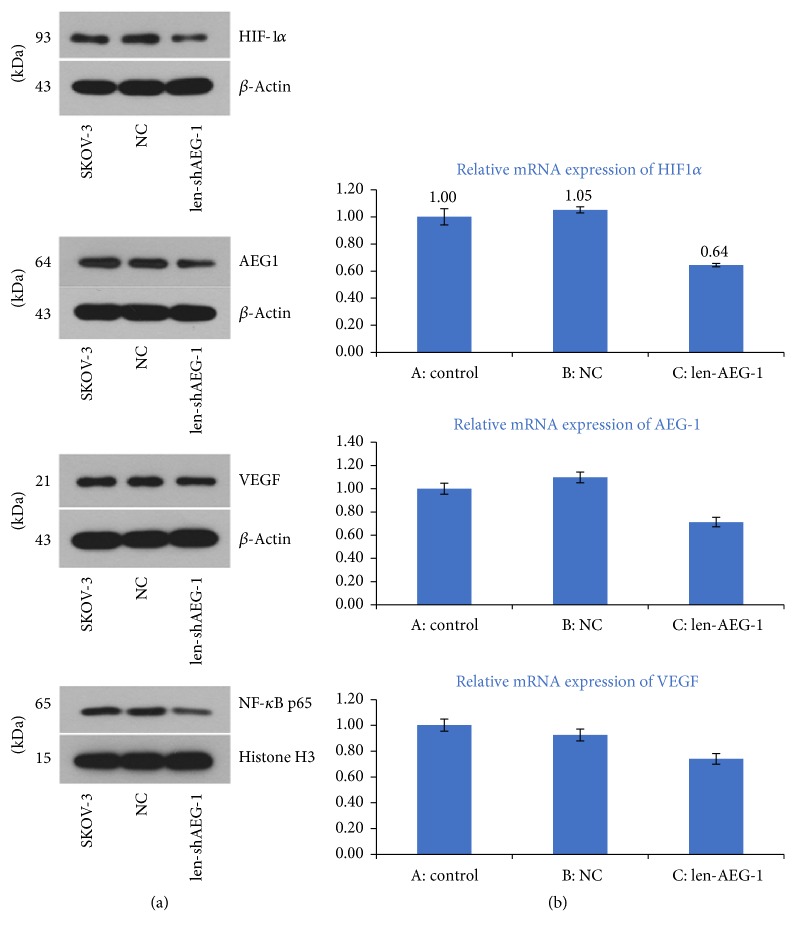
*AEG-1, HIF-1α, NF-κB, and VEGF expression levels in wild type and AEG-1 knockdown SKOV3 cells cultured under hypoxic conditions*. (a) After AEG-1 knockdown by lentiviral infection, AEG-1, HIF-1*α*, NF-*κ*B, and VEGF protein amounts were significantly reduced (all *p* < 0.001) as assessed by Western blotting. (b) After AEG-1 knockdown, AEG-1, HIF-1*α*, and VEGF gene expression levels were significantly reduced (all *p* < 0.001) as determined by real-time PCR. Data are mean ± SD from three independent experiments.

We found that hypoxia associated upregulation of NF-*κ*B, HIF-1*α*, and VEGF in SKOV3 cells was modulated by AEG-1. Indeed, NF-*κ*B, HIF-1*α*, and VEGF protein amounts were significantly decreased by AEG-1 silencing (Figure 5, *p* < 0.01). In addition, AEG-1 could regulate hypoxia induced transcription of HIF-1*α*, NF-*κ*B, and VEGF in ovarian cancer cells (Figure 5).

## 4. Discussion

EOC is the predominant type of ovarian carcinoma and the deadliest malignancy in females, with a very high prevalence in China. The currently available diagnostic markers are not effective enough. Therefore, more adequate markers should be identified to predict metastasis and/or recurrence in EOC patients.

AEG-1 was firstly described as a gene induced in primary human fetal astrocytes. Then, studies revealed its important roles in many aspects of cancer. Clinical and functional analyses have showed that AEG-1 could be considered a potentially crucial target in the treatment of malignant neoplasms. In this study, AEG-1 was upregulated in human EOC and significantly associated with multiple clinicopathological characteristics (Table 2) [5]. These findings suggested that AEG-1 plays important roles in tumor progression and metastasis.

HIF-1*α* has critical functions in hypoxic response of human cells, modulating hypoxia-inducible genes [4]. It is known that HIF-1*α* increases blood, oxygen, and energy supply to tumors, attenuating hypoxia, [6] and has crucial functions in cancer cell metabolism, angiogenesis, and metastasis. Meanwhile, HIF-1*α* is regulated by many factors [7].

As shown above, 62.9%, 60.0%, and 54.7% of EOC patients had elevated AEG-1, HIF-1*α*, and VEGF amounts, respectively, rates which were markedly higher than control values (Table 1). These results corroborated previous findings that AEG-1 is involved in tumor progression [1, 8–20]. According to our statistical analysis, high AEG-1 and HIF-1*α* expression levels were commonly associated with elevated metastasis (*p* < 0.01) as well as lower FIGO stage (*p* < 0.001) and degree of differentiation (*p* < 0.001), in patients with advanced ovarian carcinoma and lymph node metastasis (Table 2), as reported in previous studies [21, 22]. This suggests that AEG-1 and HIF-1*α* are potential early diagnostic markers for ovarian carcinoma.

Furthermore, we demonstrated that AEG-1 levels were obviously associated with HIF-1*α* and VEGF amounts (Table 3, *p* < 0.001). To assess intercorrelations among these factors, AEG-1, HIF-1*α*, NF-*κ*B, and VEGF mRNA and protein amounts were quantitated by quantitative real-time PCR and Western blotting, respectively, at different times in EOC cells exposed to hypoxia. Interestingly, SKOV3 cells showed significantly increased levels of the above factors after exposure to hypoxia (Figure 4), with peaks observed at 8 h (*p* < 0.001). In addition, the expression levels of NF-*κ*B and VEGF were evaluated in AEG-1 knockdown ovarian cancer cells by RT-PCR and Western blotting, respectively. After AEG-1 knockdown, HIF-1*α*, NF-*κ*B, and VEGF mRNA and protein amounts were significantly decreased in EOC SKOV3 cells cultured under hypoxic conditions (Figure 5).

As demonstrated above, HIF-1*α*, NF-*κ*B, and VEGF were downregulated after AEG-1 silencing. These findings suggest that AEG-1 induces HIF-1*α*/NF-*κ*B/VEGF signaling in hypoxic SKOV3 cells. In agreement, the significance of an AEG-1- dependent pathway was revealed in tumor-associated angiogenesis and cancer progression, with VEGF downstream of AEG-1 [15]. Considering the crosstalk between AEG-1 and HIF-1*α*/NF-*κ*B/VEGF signaling, AEG-1 might be involved in hypoxia regulation. Consistently, tight associations of AEG-1, VEGF, and HIF-1*α* levels in EOC were found in this work. However, whether combining such prognostic biomarkers would improve prognosis in EOC requires further assessment.

## 5. Conclusions

*Overall*, the above findings indicated HIF-1*α* and AEG-1 are critical angiogenic markers and constitute potential prognostic factors in EOC exposed to a hypoxic environment. Combined expression analysis of AEG-1, HIF-l*α*, and VEGF may help determine the degree of malignancy, metastasis, and prognosis in EOC. Further studies and evidence are required to evaluate whether the HIF-1*α* and AEG-1 proteins might help predict unfavorable biological behaviors and/or constitute targets to determine the patients benefiting from antiangiogenic agents. Additional prospective studies are warranted to confirm the current findings.

## Figures and Tables

**Table 1:** AEG-1, VEGF, and HIF-1*α* levels in ovarian cancer and normal tissue specimens.

Group	Cases (*N*)	AEG-1 high expression *n *(%)	*p*	VEGF high expression *n *(%)	*p*	HIF-1*α* high expression *n *(%)	*p*
EOC tissues	170	107 (62.9)	<0.001	93 (54.7)	<0.001	102 (60.0)	<0.001
Normal ovary	40	2 (5)		4 (10)		2 (5)	

**Table 2:** Associations of HIF-1*α* and AEG-1 levels with clinicopathologic features of EOC patients.

Variable	Cases (*N*)	AEG-1 high levels		HIF-1*α* high levels	
		*n *(%)	*p*	*n *(%)	*p*
Age			0.271		0.697
≤55	63	43 (68.3)		39 (61.9)	
>55	107	64 (59.8)		63 (58.9)	
Metastasis			0.005		0.001
Negative	28	11 (39.3)		9 (32.1)	
Positive	142	96 (67.6)		93 (65.5)	
Histological type			0.002		0.006
Serous	111	79 (71.2)		75 (67.6)	
Others	59	28 (47.5)		27 (45.8)	
Grade			0.644		0.035
G1-2	101	65 (64.4)		54 (53.5)	
G3	69	42 (60.9)		48 (69.6)	
FIGO stage			0.000		0.001
I-II	45	19 (42.2)		18 (40.0)	
III-IV	125	88 (70.4)		84 (67.2)	
Residual tumor (cm)			0.000		0.000
≤1	81	37 (45.7)		34 (42.0)	
>1	89	70 (78.7)		68 (76.4)	
Ascites (ml)			0.008		0.019
≤500	62	31 (50.0)		30 (48.4)	
>500	108	76 (70.4)		72 (66.7)	
Menopausal status			0.251		0.094
No	55	38 (69.1)		38 (69.1)	
Yes	115	69 (60.0)		64 (55.7)	
Preoperative CA125 level (U/ml)			0.913		0.820
≤35	14	9 (64.3)		8 (57.1)	
>35	156	98 (62.8)		94 (60.3)	
Chemo.			0.261		0.648
No	61	35 (57.4)		38 (62.3)	
Yes	109	72 (66.1)		64 (58.7)	

**Table 3:** Associations of HIF-1*α*, VEGF, and AEG-1 levels in EOC.

	Expression		
	AEG-1 versus VEGF	AEG-1 versus HIF-1*α*	VEGF versus HIF-1*α*
*p*	<0.001	<0.001	0.01

## References

[1] Kang D. C., Su Z. Z., Sarkar D., Emdad L., Volsky D. J., Fisher P. B. (2005). Cloning and characterization of HIV-1-inducible astrocyte elevated gene-1, AEG-1. *Gene*.

[2] Huang Y., Li L.-P. (2014). Progress of cancer research on astrocyte elevated gene-1/Metadherin. *Oncology Letters*.

[3] Su Z.-Z., Kang D.-C., Chen Y. (2002). Identification and cloning of human astrocyte genes displaying elevated expression after infection with HIV-1 or exposure to HIV-1 envelope glycoprotein by rapid subtraction hybridization, RaSH. *Oncogene*.

[4] de Vallière C., Cosin-Roger J., Simmen S. (2016). Hypoxia Positively Regulates the Expression of pH-Sensing G-Protein–Coupled Receptor OGR1 (GPR68). *Cellular and Molecular Gastroenterology and Hepatology*.

[5] Meng F., Luo C., Ma L., Hu Y., Lou G. (2011). Clinical significance of astrocyte elevated gene-1 expression in human epithelial ovarian carcinoma. *International Journal of Gynecological Pathology*.

[6] Yasuda S., Arii S., Mori A. (2004). Hexokinase II and VEGF expression in liver tumors: correlation with hypoxia-inducible factor-1*α* and its significance. *Journal of Hepatology*.

[7] Semenza G. L. (2003). Targeting HIF-1 for cancer therapy. *Nature Reviews Cancer*.

[8] Semenza G. L. (2010). Defining the role of hypoxia-inducible factor 1 in cancer biology and therapeutics. *Oncogene*.

[9] Hu G., Chong R. A., Yang Q. (2009). MTDH Activation by 8q22 Genomic Gain Promotes Chemoresistance and Metastasis of Poor-Prognosis Breast Cancer. *Cancer Cell*.

[10] Sarkar D., Emdad L., Lee S.-G., Yoo B. K., Su Z.-Z., Fisher P. B. (2009). Astrocyte elevated gene-1: Far more than just a gene regulated in astrocytes. *Cancer Research*.

[11] Hu G., Wei Y., Kang Y. (2009). The multifaceted role of MTDH/AEG-1 in cancer progression. *Clinical Cancer Research*.

[12] Lee S.-G., Su Z.-Z., Emdad L., Sarkar D., Fisher P. B. (2006). Astrocyte elevated gene-1 (AEG-1) is a target gene of oncogenic Ha-ras requiring-phosphatidylinositol 3-kinase and c-Myc. *Proceedings of the National Acadamy of Sciences of the United States of America*.

[13] Emdad L., Sarkar D., Su Z.-Z. (2007). Corrigendum to “Astrocyte elevated gene-1: Recent insights into a novel gene involved in tumor progression, metastasis and neurodegeneration” [Pharmacol. Ther., 114 (2007) 155-170] (DOI:10.1016/j.pharmthera.2007.01.010). *Pharmacology & Therapeutics*.

[14] Sarkar D., Eun S. P., Emdad L., Lee S.-G., Su Z.-Z., Fisher P. B. (2008). Molecular basis of nuclear factor-*κ*B activation by astrocyte elevated gene-1. *Cancer Research*.

[15] Emdad L., Lee S.-G., Su Z. Z. (2009). Astrocyte elevated gene-1 (AEG-1) functions as an oncogene and regulates angiogenesis. *Proceedings of the National Acadamy of Sciences of the United States of America*.

[16] Li J., Yang L., Song L. (2009). Astrocyte elevated gene-1 is a proliferation promoter in breast cancer via suppressing transcriptional factor FOXO1. *Oncogene*.

[17] Su P., Zhang Q., Yang Q. (2010). Immunohistochemical analysis of Metadherin in proliferative and cancerous breast tissue. *Diagnostic Pathology*.

[18] Li J., Zhang N., Song L.-B. (2008). Astrocyte elevated gene-1 is a novel prognostic marker for breast cancer progression and overall patient survival. *Clinical Cancer Research*.

[19] Yoo B. K., Emdad L., Su Z.-Z. (2009). Astrocyte elevated gene-1 regulates hepatocellular carcinoma development and progression. *The Journal of Clinical Investigation*.

[20] Yu C., Chen K., Zheng H. (2009). Overexpression of astrocyte elevated gene-1 (AEG-1) is associated with esophageal squamous cell carcinoma (ESCC) progression and pathogenesis. *Carcinogenesis*.

[21] Daponte A., Ioannou M., Mylonis I. (2008). Prognostic significance of hypoxia-inducible factor 1 alpha(HIF-1alpha) expression in serous ovarian cancer: An immunohistochemical study. *BMC Cancer*.

[22] Marín-Hernández A., Gallardo-Pérez J. C., Ralph S. J., Rodríguez-Enríquez S., Moreno-Sánchez R. (2009). HIF-1*α* modulates energy metabolism in cancer cells by inducing over-expression of specific glycolytic isoforms. *Mini-Reviews in Medicinal Chemistry*.

